# Coexistent pleural effusion is found to be associated with aggravated subclinical myocardial injury in systemic lupus erythematous using cardiovascular magnetic resonance imaging

**DOI:** 10.3389/fimmu.2024.1504624

**Published:** 2024-11-25

**Authors:** Yang Zhi, Tian-yue Zhang, Yong Zhu, Hao Zou, Yi You, Miao Wen, Zhong Wang, Liang-chao Gao, Fu Bing, Shu-yue Pan

**Affiliations:** ^1^ Department of Radiology, Chengdu Fifth People’s Hospital, Chengdu, China; ^2^ Department of Rheumatology and Immunology, Chengdu Fifth People’s Hospital, Chengdu, China

**Keywords:** cardiovascular magnetic resonance imaging, late gadolinium enhancement, strains, systemic lupus erythematous, T1 mapping, T2 mapping

## Abstract

**Objective:**

Pleural effusion (PE) is a common pulmonary manifestation in patients with systemic lupus erythematosus (SLE), and is associated with disease activity. However, little is known regarding the additive effects of PE on cardiac function. Therefore, this study aimed to investigate multi-parameter cardiovascular magnetic resonance imaging (CMR) findings in SLE patients with PE and to explore whether cardiac involvement is associated with PE.

**Methods:**

Patients with SLE and age-matched/sex-matched healthy controls were included in this study. Patients with SLE were diagnosed according to the 2019 European League Against Rheumatism/American College of Rheumatology classification criteria. Moreover, the PE diagnosis was based on computed tomography, and the height of the effusion was > 5 mm. All enrolled individuals underwent CMR imaging, including cine and late gadolinium enhancement (LGE), T1, and T2 mapping imaging. The left and right ventricular function, LGE, T1, extracellular volume (ECV), and T2 values were evaluated.

**Results:**

A total of 111 patients with SLE were enrolled, of whom 26 (23.42%) had PE. White cell count, hemoglobin, CRP, ESR, and lactate dehydrogenase levels were higher in SLE patients with PE than in SLE patients without PE (P<0.05). LGE was more prevalent in SLE patients with PE compared with those without PE (P<0.001). In addition, Native T1 (1348 ± 65 ms vs. 1284 ± 67 ms vs. 1261 ± 41 ms; P<0.001), ECV (31.92 ± 4.16% vs. 28.61 ± 3.60% vs. 26.54 ± 2.94%; P<0.001), and T2 (44.76 ± 3.68 ms vs. 41.96 ± 3.62 ms vs. 39.21 ± 2.85 ms; P<0.001) values were high in SLE patients with PE, intermediate in SLE patients without PE, and the lowest in the control group. Linear regression analysis demonstrated that PE was independently associated with LGE (β=0.329; P<0.05), T1 (β=0.346; P<0.05), ECV (β=0.353; P<0.05), and T2 (β=0.201; P<0.05).

**Conclusions:**

SLE patients with PE have a higher prevalence of LGE and more diffuse myocardial fibrosis and edema than SLE patients without PE. Moreover, PE is associated with increased diffuse interstitial fibrosis and edema.

## Introduction

Systemic lupus erythematosus (SLE) is an autoimmune disease that mainly affects women and tends to involve more than one system (1). In many SLE patients, pleural effusion (PE) is a common pulmonary manifestation with a prevalence ranging from 16% to 50% and has been linked to disease activity (2, 3). Previous studies have demonstrated that SLE patients with a higher level of disease activity have an increased risk of cardiovascular disease (4–6). Cardiac involvement is the leading cause of death in patients with SLE, even in the early stages of the disease (7–9). Considering that SLE patients with PE often reflect disease activity and are likely to have cardiac involvement in clinical practice, this finding suggests that an assessment of cardiac involvement in these patients is crucial (10).

Cardiovascular magnetic resonance imaging (CMR) is a promising technique that allows the non-invasive characterization of myocardial tissue characteristics using late gadolinium enhancement (LGE), and T1 and T2 mapping (11–13). Previous studies have shown that T1 and extracellular volume (ECV) values, an indirect measure of myocardial interstitial fibrosis, and T2 values, a parameter of edema, are usually present in patients with SLE (14, 15). According to recent studies, patients with SLE have significantly higher T1, ECV, and T2 values, indicating subclinical myocardial damage (16–18). Although disease activity correlates with myocardial damage in patients with SLE, little is known about the additive effect of PE on the heart, including fibrosis and edema (4). Therefore, this study aimed to investigate multi-parameter CMR findings in SLE patients with PE and to explore whether cardiac involvement is associated with PE.

## Methods

### Study population

This prospective study was approved by our Institutional Research Ethics Board, and written informed consent was obtained. All included participants were continuously enrolled at Chengdu Fifth People’s Hospital between January 2021 and March 2024. CMR, chest computed tomography (CT), and echocardiography were performed in patients diagnosed with SLE according to the 2019 European League Against Rheumatism/American College of Rheumatology classification criteria (19). The exclusion criteria were as follows: (1) age < 18 years; (2) known cardiomyopathies, including hypertrophic cardiomyopathy, dilated cardiomyopathy, amyloidosis, and cardiac sarcoidosis; (3) severe valvular disease; and (4) poor image quality or incomplete clinical record. PE diagnosis was based on CT of the chest, and SLE patients with a height of effusion > 5 mm were enrolled into the SLE patients with PE cohort (20, 21). Therefore, SLE patients were divided into two cohorts: SLE patients without PE and SLE patients with PE. Age- and sex-matched healthy controls without known myocardial disease or suspected myocarditis on CMR were recruited as the control group.

Clinical records, including age, sex, disease activity, hepatic and renal function, and medications, were obtained from electronic medical records.

### CMR image acquisition

CMR imaging was performed using a Siemens Vida 3.0T MRI system with a 32-channel body coil and electrocardiogram-gating. Cine, pre-contrast T1 mapping, LGE imaging, and post-contrast T1 mapping were obtained with short-axis and 4-chamber views while the patient held their breath. Cine images were obtained using a balanced steady-state free precession (SSFP) sequence (repetition time (TR): 39.12 ms; echo time (TE): 1.43 ms; flip angle (FA): 80°; field of view (FOV): 420 mm; matrix: 256×199; phase: 25) in long-axis 2-chamber, 3-chamber, 4-chamber, and short-axis 2-chamber (8 mm slice thickness and 2 mm gap). Pre- and post-T1 mapping were acquired using a modified Look-Locker inversion recovery (MOLLI) sequence before and 10-15 minutes after an intravenous administration of 0.2 mmol/Kg of gadolinium diethylenetriamine penta-acetic acid (Gd-DTPA) (MultiHance; Bracco) with the following parameters: TR: 257.3 ms; TE: 0.95 ms; FA: 35°; FOV: 420 mm; matrix: 256×144; bandwidth: 1085 Hz/Px; echo spacing: 2.24 ms. T2 mapping imaging was obtained before Gd-DTPA was injected with the following parameters: TR: 224.8 ms; TE: 1.22 ms; FA: 12°; FOV: 420 mm; matrix: 192×116; bandwidth: 1184 Hz/Px; echo spacing: 2.94 ms. LGE images were obtained 10-15 minutes after Gd-DTPA injection using the phase-sensitive inversion recovery (PSIR) sequence with the following parameters: TR: 740 ms; TE: 1.06 ms; FA: 40°; FOV: 420 mm; matrix: 256×144.

### CMR analysis

All CMR images were analyzed using offline software (CMR42, v. 5.15.4, Circle Cardiovascular Imaging, Calgary, Canada) by an investigator with 5 years of CMR experience. Short-axis images were loaded into the functional SAX module, the endocardial and epicardial borders were automatically traced in the LV end-diastole and end-systole phases, and LV and RV functional parameters, including LV ejection fraction (LVEF), LV end-diastolic (LVEDV), LV end-systolic (LVESV), LV stroke volume (LVSV), LV mass, RV ejection fraction (RVEF), RV end-diastolic (RVEDV), RV end-systolic (RVESV), and RV stroke volume (RVSV), were calculated. Papillary muscles were calculated as the LV volume. LV strains, including global radial strain (GRS), global circumferential strain (GCS), and global longitudinal strain (GLS), were measured using the 3D feature-tracking module with 2-chamber, 4-chamber long-axis, and short-axis cine images.

Pre- and post-contrast T1 mapping were loaded into the tissue T1 mapping module to obtain the Native T1 and ECV values. ECV values were calculated using the formula described in a previous study (22). Hematocrit was obtained within 1 week of the CMR scan. T2 mapping images were analyzed using the tissue T2 mapping module, and the mean T2 values for the global LV myocardium were obtained by manually drawing the endocardial and epicardial borders. The presence of LGE was visually assessed by an investigator blinded to the clinical data.

### Statistical analysis

All data were analyzed using GraphPad Prism 7 (Version 7.00, GraphPad Software, Inc.) or SPSS (Version 23.0, Released 2015, IBM Corp). Continuous variables were tested using the D’Agostino and Pearson normality test and expressed as mean ± SD or median (interquartile range, Q25–75). Categorical data were expressed as numbers and percentages. The unpaired t-test, Mann-Whitney U test, or chi-square test were performed to compare the SLE patients and controls as appropriate. In addition, comparisons between three groups (SLE patients with PE, SLE patients without PE, and controls) were conducted using a one-way analysis of variance (ANOVA) or Kruskal-Wallis test on the clinical and CMR variables. Pearson’s correlation was performed to assess the relationship between T1, ECV, and T2 values. Univariable and multivariable linear regressions were used to identify independent factors of tissue characteristic parameters. Variables with P-values less than 0.10 in the univariate linear regression analysis were included in the multivariate linear regressions. A P-value less than 0.05 was considered significant.

## Results

### Patient characteristics

A total of 123 patients with SLE who underwent CMR imaging were enrolled in the study from our hospital between January 2021 and March 2023. Based on the inclusion and exclusion criteria, 12 patients with SLE were excluded (six SLE patients underwent repeated CMR scans, one SLE patient had hypertrophic cardiomyopathy, two SLE patients had myocardial infarction, two SLE patients had severe valvular disease, and one SLE patient was aged < 18 years). Finally, 111 patients with SLE were included, and the majority of the patients were female (87.38%). Of the 111 SLE patients, 26 (23.42%) had PE, and the remaining 85 (76.58%) were classified as having SLE without PE. In addition, we recruited 24 age- and sex-matched healthy individuals with no significant systemic or cardiovascular diseases as the control group. The baseline characteristics of the study cohort are presented in **Table 1**. For patients with SLE, there was no difference in sex distribution between those with and without PE (P>0.05). Moreover, there was no significant difference in age among the three groups (P>0.05).

**Table 1:** Baseline characteristics of the patients with SLE with or without PE.

		SLE without PE (n=85)	SLE with PE (n=26)	P value*
Age, years	45.08±14.76	43.66±14.36	41.23±13.39	0.538
Female, n (%)	17(70.83)	74(80.06)	23(88.46)	0.125
Disease duration, years	-	2.00(0.20,7.00)	2.00(0.20,8.50)	0.906
BMI	–	21.79±3.03	22.17±4.07	0.628
Systolic blood pressure (mmHg)	-	124.2±15.65	118.4±17.38	0.074
Diastolic blood pressure (mmHg)	–	80.69±13.16	74.04±14.81	**0.033**
Clinical				
Laboratory results				
White cell count (×10^9^/L)	-	5.33±2.79	7.24±3.29	**0.005**
Neutral lymphocytes (%)	–	61.75(2.12, 80.70)	77.30(67.75, 84.55)	0.056
Haemoglobin (g/dl)	-	112.90±22.17	99.94±19.34	**0.009**
Hematocrit (%)	–	35.81±8.61	31.60±6.00	**0.012**
CRP (mg/L)	-	3.00(1.20, 11.00)	16.90(2.00, 52.30)	**0.002**
ESR (mm/1^st^ hour)	–	32.00(12.00, 67.00)	55.00(31.50, 88.00)	**0.003**
Urinary protein positive, n (%)	-	31(39.24)	18(72.00)	**0.005**
Creatinine (mg/mL)	–	61.95±22.75	73.10±34.99	0.239
Urea nitrogen (pg/mL)	-	5.73(4.24, 7.79)	5.84(4.36, 8.62)	0.797
Serum albumin (g/L)	–	37.86±6.69	30.68±7.77	**<0.001**
Glutamic-pyruvic transaminase (U/L)	-	24.00(15.00, 38.00)	23.50(12.25, 48.75)	0.983
Glutamic oxaloacetic transaminase (U/L)	–	32.01±23.00	47.00±41.22	0.336
Blood glucose (mg/dL)	-	5.36±1.20	4.95±0.90	0.172
Total cholesterol (mmol/L)	–	4.13±0.96	4.63±1.43	0.159
Triglyceride (mmol/L)	-	1.47(1.09, 2.01)	1.79(1.24, 2.74)	0.179
HDL-cholesterol (mmol/L)	–	1.03±0.38	1.17±0.41	0.149
Blood calcium (mmol/L)	-	2.15±0.13	1.99±0.18	**<0.001**
Blood phosphorus (mmol/L)	–	1.13±0.24	1.22±0.23	0.119
Blood potassium (mmol/L)	-	3.76±0.46	3.85±0.52	0.233
Blood sodium (mmol/L)	–	141.80±4.91	140.10±4.03	**0.019**
Serum C3 (mg/L)	-	0.76±0.28	0.70±0.35	0.259
Serum C4 (mg/L)	–	0.12±0.08	0.12±0.07	0.687
ANA positive, n (%)	-	76(92.68)	20(89.96)	0.407
Anti-dsDNA positive, n (%)	–	38(48.72)	13(56.52)	0.636
ANCA positive, n (%)	-	27(35.06)	8(34.78)	>0.999
ACA-IgG positive (U/L)	–	16.47±28.37	9.86±9.06	0.728
Creatine Kinase (U/L)	-	40.00(26.75, 70.75)	53.00(24.00, 209.0)	0.091
CK-MB (U/L)	–	10.00(8.00, 13.00)	11.00(9.00, 16.75)	0.248
Lactate dehydrogenase (U/L)	-	211.0(162.0, 260.0)	261.0(207.3, 419.0)	**0.001**
Medicine, n (%)				
Prednisone	–	73(90.12)	24(100)	0.193
Hydroxychloroquine	-	70(86.42)	18(75)	0.211
Methotrexate	–	4(4.94)	1(4.17)	0.507
Azathioprine	-	3(3.7)	2(8.33)	0.321
Rebamipide	–	28(34.57)	8(33.33)	>0.999

Values are mean ± SD, number (%), or median (25th-75th percentile). Numbers in boldface indicate P values <0.05.

*P value across the 3 groups.

SLE, systemic lupus erythematous; PE, pleural effusion; BMI, body mass index; CRP, c-reactive protein; ESR, erythrocyte sedimentation rate; HDL, high-density lipoprotein; ANA, antinuclear antibody; ANCA, anti-neutrophil cytoplasmic antibodies; ACA-IgG, human anti-cardiolipin antibody-immunoglobulin G; CK-MB, creatine kinase-MB.

Disease duration did not differ significantly between SLE patients with and without PE (P>0.05). However, immune parameters including white cell count, hemoglobin, CRP, and ESR were higher in the SLE patients in the PE group than in the SLE patients without PE (P<0.05). In addition, the prevalence of urinary protein positivity was greater in patients with SLE with PE than in those without PE (P<0.05). Compared to SLE patients without PE, lactate dehydrogenase levels were increased and blood calcium levels were reduced in SLE patients with PE (P<0.05).

### Cardiac function and LV strains

Pericardial effusion was more frequent in SLE patients with PE than those without PE (P<0.05). However, there were no significant differences in LVEDV, LVESV, LVSV, LVEF, LVCO, LV mass, RVEDV, RVESV, RVSV, RVEF, and RVCO among the three groups (P>0.05). Furthermore, there were no significant differences in GRS, GCS, and GLS between the three groups (P>0.05) (**Table 2**).

**Table 2:** Imaging characteristics of patients with SLE depending on the presence of PE.

	Control (n=24)	SLE without PE (n=85)	SLE with PE (n=26)	P value*
CMR				
HR,	73.31±13.86	74.69±15.63	74.88±16.34	0.915
Pericardial effusions	0(0)	6(7.05)	13(50)	**<0.001**
LV morphology & function				
LVEDV, ml	102.20±21.31	107.60±26.41	212.40±38.41	0.079
LVESV, ml	37.83±11.19	39.13±14.86	49.94±36.05	0.450
LVSV, ml	64.23±13.45	68.43±18.85	71.46±15.91	0.190
LVCO, ml	4.69±1.37	4.98±1.30	5.29±1.33	0.126
LVEF, %	63.05±6.43	63.71±9.80	62.20±12.48	0.560
LV mass, g	67.75±17.25	69.99±19.28	77.37±23.54	0.121
LV strain				
GRS, %	39.95±12.02	35.66±11.73	35.49±12.16	0.345
GCS, %	-18.78±2.40	-19.12±3.43	-19.15±4.28	0.439
GLS, %	-14.53±2.92	-13.83±3.83	-13.38±3.48	0.403
RV morphology & function				
RVEDV, ml	107.10±25.92	113.40±27.39	119.00±23.27	0.241
RVESV, ml	53.12±16.90	50.43±18.55	55.70±20.30	0.475
RVSV, ml	53.96±15.23	62.97±17.84	63.33±17.15	0.057
RVCO,	3.88±1.26	4.57±1.33	4.70±1.46	0.050
RVEF, %	48.41±14.01	55.86±11.52	53.72±12.52	0.073
LV myocardial LGE				
LGE present, n (%)	0(0)	27(32.14)	18(72.00))	**<0.001**
LV tissue characterization				
Native T1, ms	1261±41	1284±67	1348±65‡†	**<0.001**
ECV, %	26.54±2.94	28.61±3.60†	31.92±4.16‡†	**<0.001**
Native T2, ms	39.21±2.85	41.96±3.62†	44.76±3.68‡†	**<0.001**

*P value across the 3 groups.

†P<0.05 between individual group compared with control.

‡P<0.05 between SLE with PE group compared with SLE without PE group.

SLE, systemic lupus erythematous; PE, pleural effusion; CMR, cardiac magnetic resonance; HR, heart rate; LV, left ventricle; LVEDV, left ventricular end diastolic volume; LVESV, left ventricular end systolic volume; LVSV, left ventricular stroke volume; LVCO, left ventricular cardiac output; LVEF, left ventricular ejection fraction; GRS, global radial strain; GCS, global circumferential strain; GLS, global longitudinal strain; RV, right ventricle; RVEDV, right ventricular end diastolic volume; RVESV, right ventricular end systolic volume; RVSV, right ventricular stroke volume; RVCO, right ventricular cardiac output; RVEF, right ventricular ejection fraction; LGE, late gadolinium enhancement; ECV, extracellular volume. Numbers in boldface indicate P values <0.05.

### Scar burden, myocardial fibrosis, and edema

Among our SLE patients, 45 had LGE. LGE was more prevalent in the SLE patients with PE compared with those without PE (P<0.001).

**Figure 1:**
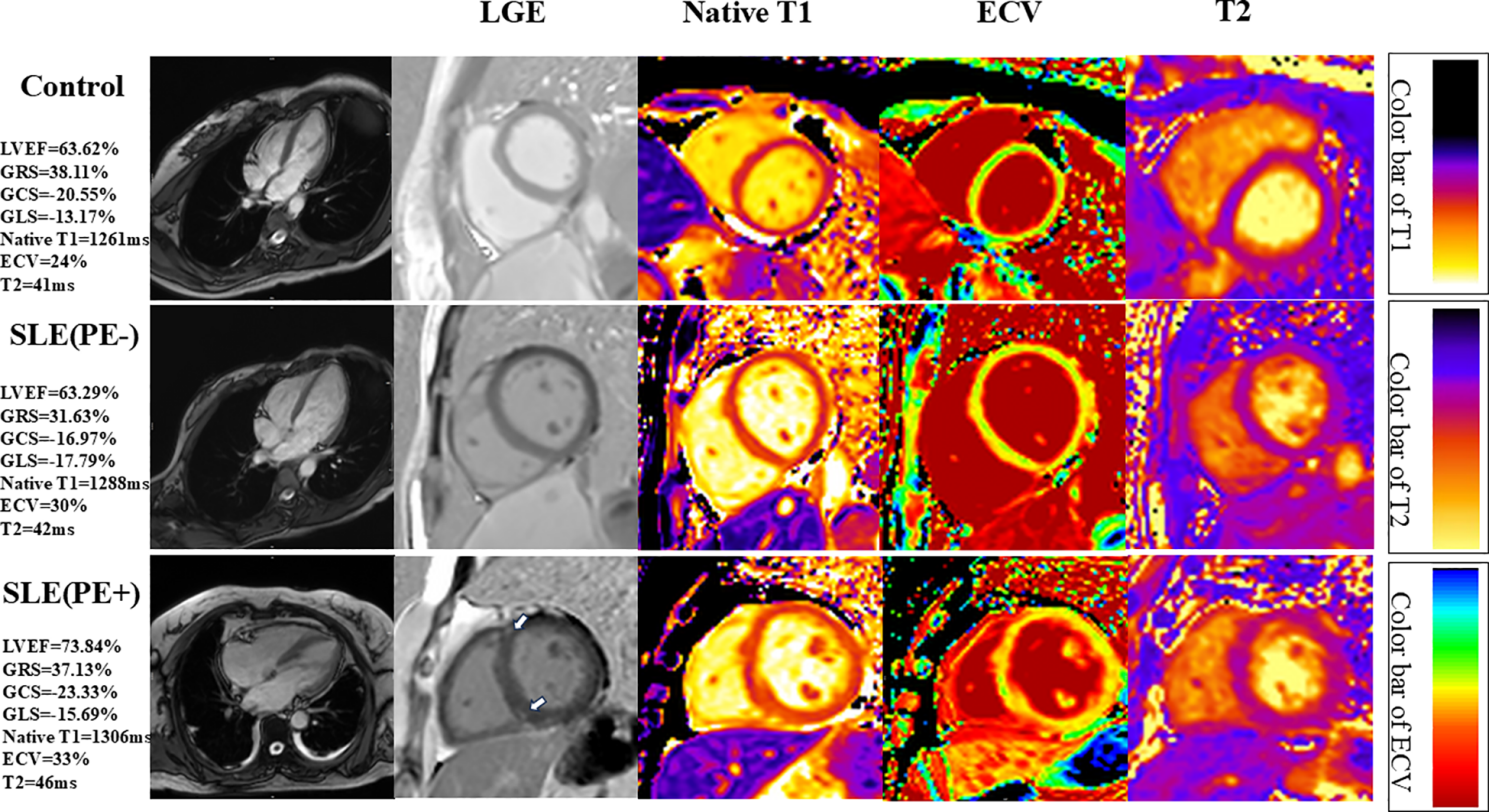
Representative short-axis cardiovascular magnetic resonance (CMR) images of systemic lupus erythematosus (SLE) patients with or without pleural effusion (PE) and controls. Please note that the SLE patients with PE have LGE at right ventricular insertion points (narrow arrows). LVEF, left ventricular ejection fraction; GRS, global radial strain; GCS, global circumferential strain; GLS, global longitudinal strain.

Native T1 (1348 ± 65 ms vs. 1284 ± 67 ms vs. 1261 ± 41 ms; P<0.001) and ECV (31.92 ± 4.16% vs. 28.61 ± 3.60% vs. 26.54 ± 2.94%; P<0.001) values were highest in the SLE patients with PE, intermediate in SLE patients without PE and the lowest in the control group (**Figure 1**; **Table 2**).

Moreover, edema, indicated by the T2 values, was greater in SLE patients with PE, intermediate in SLE patients without PE, and lowest in the control group (44.76 ± 3.68 ms vs. 41.96 ± 3.62 ms vs. 39.21 ± 2.85 ms; P<0.001) (**Figure 1**; **Table 2**).

### Correlation of Native T1 and ECV with increased T2 values

**Figure 2:**
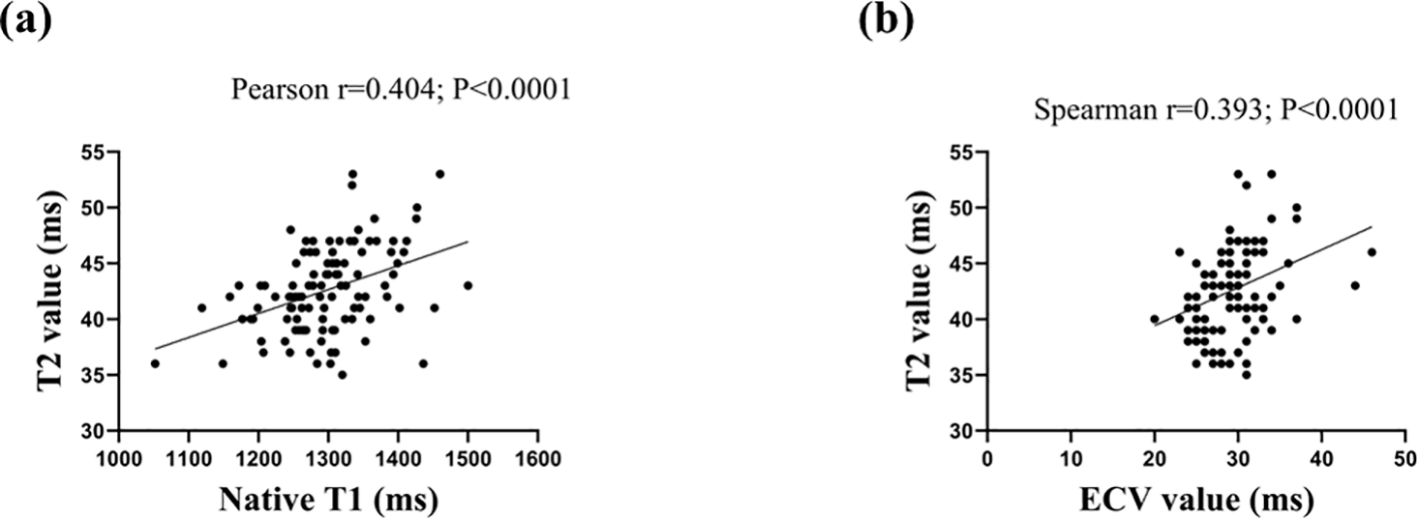
Scatter plots depicting the relationship between T2 and Native T1 values (2A) and extracellular volume (ECV) value (2B).

The analyses of the relationships between Native T1, ECV, and T2 values are shown in **Figure 2**. In the SLE patients, Native T1 was moderately associated with T2 (Pearson r=0.404; P<0.001). In addition, there was a positive correlation between ECV and increased T2 values in patients with SLE (Spearman’s r=0.393; P<0.001).

### Relationship between clinical parameters and T1 mapping, T2 mapping, and LGE in SLE patients

As demonstrated in **Table 3**, univariate and multivariate linear regressions showed that PE was independently correlated with LGE (β=0.329; P<0.05), T1 (β=0.346; P<0.05), ECV (β=0.353; P<0.05), and T2 values (β=0.201; P<0.05). Moreover, age was independently associated with Native T1 (β=-0.273; P<0.05). In addition, hemoglobin (β=-0.255; P<0.05) and HDL cholesterol (β=0.219; P<0.05) were independently associated with T2 values.

**Table 3:** Linear regression analysis of the LV LGE and T1 and T2 mapping in the patients with SLE.

	LGE		Native T1		ECV	T2	
	Univariable β	Multivariable β, R2=0.164	Univariable β	Multivariable β, R2=0.319	Univariable β	Univariable β	Multivariable β, R2=0.173
Male	-0.043		-0.118		-0.058	-0.111	
Age	-0.081		-0.297*	-0.273*	-0.132	-0.230*	-0.174
Disease duration	0.022		0.114		0.127	-0.027	
BMI	-0.130		0.006		0.003	0.052	
Systolic blood pressure	-0.178**	-0.082	0.058		-0.099	0.116	
Diastolic blood pressure	-0.166**	-0.040	0.022		-0.046	0.094	
White cell count	0.009		-0.123		-0.121	-0.964	
Neutral lymphocytes	0.044		-0.058		-0.081	-0.030	
Haemoglobin	0.079		-0.063		-0.060	-0.187**	-0.225*
Hematocrit	0.115		-0.005		0.010	-0.077	
CRP	0.076		-0.168**	-0.139	0.062	-0.121	
ESR	-0.097		-0.176**	-0.112	-0.041	-0.058	
Creatinine	0.088		-0.120		-0.008	0.060	
Urea nitrogen	-0.063		-0.081		-0.101	-0.006	
Serum albumin	-0.079		0.067		-0.041	0.003	
Glutamic-pyruvic transaminase	0.073		0.029		-0.010	-0.021	
Glutamic oxaloacetic transaminase	0.154		0.015		0.069	-0.047	
Blood glucose	-0.034		0.037		0.068	0.015	
Total cholesterol	0.036		-0.038		0.050	0.086	
Triglyceride	0.045		-0.028		-0.035	-0.102	
HDL-cholesterol	-0.021		0.126		0.077	0.180**	0.219*
Serum C3	-0.057		0.036		0.115	0.064	
Serum C4	0.027		-0.021		0.057	0.065	
Creatine Kinase	0.106		-0.031		0.012	-0.019	
CK-MB	-0.072		-0.078		0.006	-0.052	
Lactate dehydrogenase	0.192**	0.123	-0.011		0.081	-0.021	
HR	-0.032		0.029		-0.060	-0.064	
LVEDV	-0.019		-0.230*	-0.054	0.025	-0.032	
LVESV	0.032		-0.126		0.087	-0.037	
LVEF	0.026		-0.001		-0.077	0.053	
LV mass	-0.065		-0.272*	-0.074	0.039	0.005	
RVEDV	-0.054		-0.252*	-0.101	-0.040	-0.149	
RVESV	-0.260		-0.158		0.012	-0.164**	
RVEF	-0.005		-0.003		-0.061	0.089	
PE	0.340*	0.329*	0.378*	0.346*	0.353*	0.305*	0.201*

LV, left ventricular; LGE, late gadolinium enhancement; SLE, systemic lupus erythematous; ECV, extracellular volume; BMI, body mass index; CRP, c-reactive protein; ESR, erythrocyte sedimentation rate; HDL, high-density lipoprotein; CK-MB, creatine kinase-MB; HR, heart rate; LV, left ventricle; LVEDV, left ventricular end diastolic volume; LVESV, left ventricular end systolic volume; LVEF, left ventricular ejection fraction; RVEDV, right ventricular end diastolic volume; RVESV, right ventricular end systolic volume; RVEF, right ventricular ejection fraction; PE, pleural effusion; *P < 0.05; **P < 0.10. Numbers in boldface indicate P values <0.05.

## Discussion

This study indicated that one-fifth of patients with SLE displayed PE. Moreover, patients with SLE with PE had a high prevalence of LGE. SLE patients with PE had higher Native T1, ECV, and T2 values compared with those without PE. However, GRS, GCS, and GLS were similar in patients with SLE with or without PE.

SLE is characterized as a chronic autoimmune disease that involves one or more organs, including the lungs, kidneys, joints, and cardiac system (23). Studies have shown that adverse cardiovascular events are the main cause of death among SLE patients (24, 25). Furthermore, the lungs are often involved and the most common manifestation is PE (26). In our study, nearly one-fifth of the patients with SLE had PE. Several studies have shown that SLE-induced PE is significantly associated with disease activity (10, 27). Moreover, our research also confirmed that immune parameters, such as white cell count, hemoglobin, and hematocrit, which usually represent disease activity, were higher in SLE patients with PE than in SLE patients without PE. Therefore, patients with SLE and PE should be monitored carefully.

SLE patients with PE had significantly higher CPR and ESR levels than SLE patients without PE. This may indicate that SLE patients with PE have higher disease activity than SLE patients without PE (10). Moreover, PE is thought to be a manifestation of active SLE (28). A previous study showed that SLE patients with active disease, even those with new-onset SLE, were likely to have myocardial interstitial fibrosis and edema even when their ejection function was normal (22). Our study also found that although our SLE patients exhibited changes in myocardial interstitial fibrosis, their left ventricular strain was normal. Therefore, clinicians cannot rule out the presence of myocardial injury in patients with SLE with normal ejection function.

To the best of our knowledge, this is the first study to investigate multiparameter CMR findings in SLE patients with PE. In the present study, the LV ejection fraction was normal in SLE patients with PE and their native T1 value was elevated. Our results confirmed that T1 and ECV values are more effective than CMR strains in detecting early myocardial injury in patients with SLE. A previous study showed that cardiomyositis is caused by immune-mediated inflammation, which leads to myocardial fibrosis, including myocardial interstitial fibrosis and focal scarring (29). Moreover, PE has been associated with a high level of disease activity in patients with SLE, which can exacerbate myocardial endothelial cell damage. In other words, SLE patients with PE also have more severe inflammation than those without PE. The more severe the inflammation, the more severe the myocardial interstitial fibrosis and edema found in patients with SLE. Patients with SLE and PE have more interstitial fibrosis and edema, leading to myocardial remodeling, which may be a potential mechanism for exacerbating subclinical cardiac injury.

In our study, CMR showed a higher incidence of LGE in SLE patients with PE than in those without PE. A previous study revealed that the presence of LGE is associated with LV myocardial dysfunction and poor outcomes in patients with SLE (30). Moreover, SLE patients with PE have a heavier burden of focal LGE, compared with those without PE, indicating that these patients may require more attention and treatment (31).

T1 and T2 mapping are considered highly effective non-invasive methods for early detection and diagnosis of inflammation caused by SLE (18). Similar to previous studies, the results of this study showed that SLE patients had higher native T1 and ECV values (12, 16, 18). Notably, SLE patients with PE had significantly higher native T1 and ECV values than those with SLE alone. According to our study, SLE combined with PE may adversely affect myocardial tissue, including edema, fibrosis, and infiltration. Further studies are needed to establish whether PE treatment can reduce heart damage.

Although cardiac involvement is common in SLE patients, it is often mild or asymptomatic (32, 33). Patients with SLE lack symptoms related to cardiac injury; however, myocardial edema in the myocardial tissue can be detected by CMR (11). In addition, the T2 mapping sequence based on CMR can reflect subtle changes, such as myocardial edema, in patients with SLE (34). In our study, T2 values were elevated and associated with increased T1 and ECV values in SLE patients with PE, suggesting that PE may be a potential risk factor for myocardial edema.

CMR is a technique that can detect minor changes in the myocardium such as edema and fibrosis before systolic dysfunction occurs in SLE patients (35). The recently developed T1 and T2 quantitative mapping sequences using CMR can be sensitive and effective for depicting focal and global fibrosis, edema, and inflammation (36). In SLE patients with cardiac involvement and an increase in LV structural and functional abnormalities, targeted therapeutic interventions are needed (37). Similarly, our study confirmed that SLE patients with PE had significant cardiac damage, including fibrosis and edema, compared to those without PE. Moreover, SLE patients with PE demonstrated less favorable imaging profiles, including a higher prevalence of LGE, compared with SLE patients without PE. These findings indicate that using PE as a risk factor for myocardial injury may be needed for stricter control in patients with SLE with PE. Further research should be conducted to analyze the impact of PE on the long-term prognosis of patients with SLE.

### Limitations

This study has several limitations. First, this was an observational study with a limited number of participants. To our knowledge, this study is the first to evaluate myocardial involvement in SLE patients with PE, and our observations indicate that SLE patients with PE have more severe myocardial injury than SLE patients without PE. However, owing to the small sample size, subgroup analyses were not performed. Second, although PE was associated with elevated T1, T2, and ECV, which may indicate myocardial damage, the phenotypic characteristics of fibrosis and edema, including their location and degree, were not assessed. Third, T1 and T2 mapping and LGE sequences supported the presence of myocardial fibrosis and edema in previous studies; however, this was not confirmed by myocardial pathology. Further research using animal models is required for pathological verification.

## Conclusion

In summary, PE is associated with myocardial involvement in patients with SLE, as shown by increased diffuse myocardial fibrosis and edema, and a higher prevalence of LGE in the SLE patients with PE compared to those without PE. These adverse phenotypic features may be attributable to PE, but further mechanistic research is required.

## Data Availability

The raw data supporting the conclusions of this article will be made available by the authors, without undue reservation.
